# Legal and Regulatory Challenges for Emerging Regenerative Medicine Solutions for Diabetes

**DOI:** 10.1097/TP.0000000000004797

**Published:** 2023-09-26

**Authors:** Rebecca L. Thom, Antonia J. Cronin

**Affiliations:** 1 Peter Gorer Department of Immunobiology, MRC Centre for Transplantation, King’s College London, London, United Kingdom.; 2 Nephrology and Transplantation Centre, Guy’s and St Thomas’ Hospital NHS Trust, London, United Kingdom.; University of Geneva, Department of Surgery, Geneva, Switzerland.; Diabetes Centre, Ludwig-Maximilians University Munich, Germany.; Department of Health Sciences, University of Piemonte Orientale, Novara, Italy.; IRCCS Ospedale San Raffaele, Diabetes Research Institute, Milano, Italy.; Lyon Claude Bernard University, Dept. Transplantation, Nephrology and Clinical Immunology, Lyon, France.; European Society for Organ Transplantation, Padova, Italy.; Erlenbach, Switzerland.; Accelopment Switzerland Ltd.

## Abstract

Regenerative medicine solutions for type 1 diabetes are a rapidly developing field of medical technology. To date, these solutions have been principally cell-based treatments and at present, in Europe, these therapies are regulated under European Union regulations for advanced therapy medicinal products. But now, new emerging technology combining cellular therapy with medical devices is under development. The potential of this novel hybrid model to create a bioartificial pancreas to treat type 1 diabetes is tantalizing. However, incorporating medical devices creates a further layer of regulatory complexity. This article seeks to expose the complexity of this legal and regulatory landscape and demonstrate how evolving technology could challenge the entire existing legal paradigm. We start by summarizing the status of the only established cell-based therapy—transplantation. We set out the regulation of cellular therapies, their classification, and the role of statutory bodies. We examine the bottleneck of therapies moving from bench to bedside, and we consider the additional challenges of products, which use a combination of cells and medical devices. Finally, we argue that for the potential of this rapidly growing area of technology to be realized a seismic shift in how we regulate frontier cellular therapies will be required.

## Graphical abstract

**Figure:**
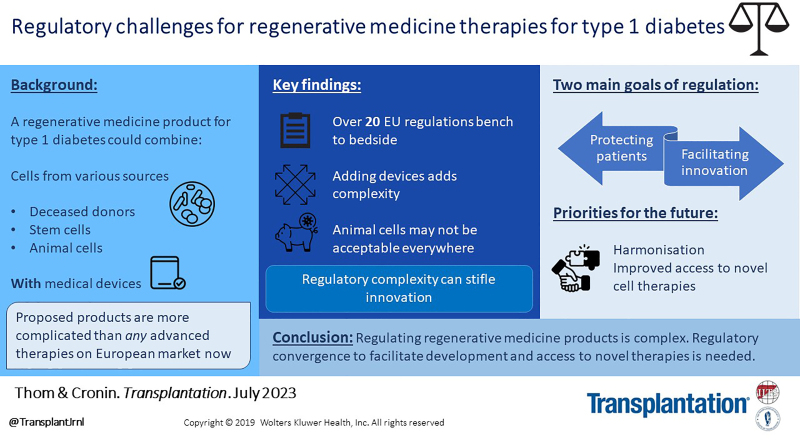

## INTRODUCTION

The treatment of type 1 diabetes has been at the cutting edge of medical innovation for over a century. Countless therapies have traversed the rocky road from hypothetical solution to routine treatment. Three principal therapeutic approaches have been used. First, pharmacological therapies, for example, synthetic insulins of various durations of action^1^ or sodium–glucose cotransporter 2 inhibitors.^2^ Second, conventional medical devices, for example, insulin pumps, glucose sensors, or mobile phone apps. Finally, therapies that use human tissues and cells, namely, whole pancreas and islet cell transplantation.

Traditionally these 3 approaches have been regulated separately under either legislation for medicinal products,^3^ medical devices,^4^ or those for transplantation.^5^ However, developments in the field of regenerative medicine have brought about the possibility of hybrid therapies in which metabolically active cells are brought together alongside medicines and devices in a single product—sometimes collectively referred to as a bioartificial pancreas.^6-8^ Such a product it is hoped may be able to return those with type 1 diabetes to a state of precise euglycemia and consequently avoid many of the damaging complications associated with this disease. However, as well as these potentially revolutionary benefits, there lie significant challenges including, as this article will argue, to the entire regulatory framework surrounding frontier cellular therapies.

This article seeks to expose the complexity of this legal and regulatory landscape, with a focus on the laws of the European Union (EU), but when appropriate bringing in examples of local variation or global perspectives. We start by summarizing the status of the only established cell-based therapy—transplantation. We then set out the regulation of cellular therapies in the EU, explaining their classification and the role of statutory bodies. We then review some of the characteristics of proposed cellular therapies for type 1 diabetes, which have been through the European assessment process and how they were classified. We examine the bottleneck of cellular therapies moving from bench to bedside and consider the part that regulatory complexity plays in this. We explore the burden of regulation and additional complications created when cellular components are combined with medical devices, the use of cells of xenogeneic origin, and how classification can impact on clinical utility. Finally, we conclude by reflecting on how these emerging regenerative medicine solutions could challenge the entire existing legal paradigm.

## MATERIALS AND METHODS

This article was synthesized by combining a review of primary legal sources, legal literature, and publicly available reports produced by the European regulator—the European Medicines Agency (EMA). Primary and secondary legislation on advanced therapy medicinal products (ATMPs) clinical research and transplantation from the European Parliament was reviewed, and a literature search was performed using the key words “regenerative medicine” on 3 main legal databases published in the English Language—LexisNexis, Westlaw, and HeinOnline.

### Transplantation—The Original Cell–based Therapy

Pancreas and islet cell transplantation represent the only currently established cell-based therapies for type 1 diabetes. In Europe, these procedures are regulated under the auspices of organ transplantation with centralized European standards dictating the requirements for determining the quality and safety of organs to be used (Directive 2010/45/EU^5^). Networks of European centers have performed 3923 whole pancreas transplants between 2015 and 2019^9^ and 2608 islet cell transplants in 1295 recipients between 2000 and 2020.^10^ Despite this extensive clinical experience, there are significant limitations to both therapies.

Whole pancreas transplant achieves insulin independence for around 83%–89% of recipients at 5 y^9,11,12^; however, 5%–10% of grafts undergo early graft loss^9,11,13^ and up to 25%–30% of patients have been reported to experience serious complications from the procedure.^11,14,15^ For islet transplantation, the complication rate is lower at 10%–14%^11,16,17^ but so are the chances of insulin independence with anywhere between 25% and 50% of recipients reported to be insulin free at 5 y.^11,16,18,19^ In both cases, long-term immune suppression with its associated risks is required. Moreover, there is an international shortage of appropriate donor pancreases, which has further limited the reach of these therapies. Combined, these factors mean that such treatments are currently reserved for only those with severe complications of diabetes—hypoglycemic unawareness, suboptimal glycemic control despite maximal medical input, and renal failure.^11,20^ The global need for new therapies without these risks and limitations is the driving force behind research to develop new cell-based treatments.

### Regulating Novel Cellular Therapies

This intensification of interest in developing regenerative therapies has brought with it enhanced scrutiny of the regulatory environment in which they exist. There is considerable variation globally in the definitions, nomenclature, classification, and regulation of cellular-based therapies. This heterogeneity is of sufficient concern that in 2021 the World Health Organization began a consultation with the expressed goal to “promote regulatory convergence for GTPs (cell and gene therapy products) to facilitate development and access to these novel products for patients in *all* regions of the world.”^21^ For the 27 countries of the EU, the European Parliament determines the type and scope of regulations, which are then implemented and enforced by the regulator—the EMA. However, even in the EU, in which there is a relative uniformity in approach, cellular products are subject to a complex matrix of local, national, and supranational rules and regulations.

#### Advanced Therapy Medicinal Products

In the EU, cellular and gene therapies are collectively known as ATMPs. Any new regenerative medicine therapy for diabetes is highly likely to fall into this classification first introduced in 2007.^22^ Historically, the majority of ATMPs have been highly targeted anticancer treatments aimed at a relatively small number of specific recipients.^23^ Gene therapies such as Libmeldy for metachromatic leukodystrophy (MLD), famous for being “the most expensive drug in the world,”^24^ are targeted at an even more limited patient group. Whereas, with approximately 9 million people living with type 1 diabetes globally^25^ and over 30 000 newly diagnosed type 1 diabetics each year in Europe alone,^26^ a successful product targeted at this group could dwarf the existing market for ATMPs. This potential has not been lost on researchers. There are currently over 300 trials registered on ClinicalTrials.gov for “diabetes mellitus type 1,” which match the key words “cell therapy.”^27^

#### ATMP Classification

ATMPs are subdivided into 4 groups:

- Gene therapy medicinal products—which “contains or consists of a recombinant nucleic acid … with a view to regulating, repairing, replacing, adding, or deleting a genetic sequence”^28^
- Somatic cell therapy products (sCTMPs)—which “contains or consists of cells or tissues that have been subject to substantial manipulation so that biologic characteristics, physiological functions, or structural properties relevant for the intended clinical use have been altered, or of cells or tissues that are not intended to be used for the same essential function(s) in the recipient and the donor”^28^
- Tissue engineered products—these contain engineered “tissues or cells with the aim to regenerate, repair, or replace a human tissue”^29^
- Combined ATMP—which meets the definition of any of these 3 but also contains 1 or more medical devices or active implantable medical devices^28,29^

**FIGURE 1.**
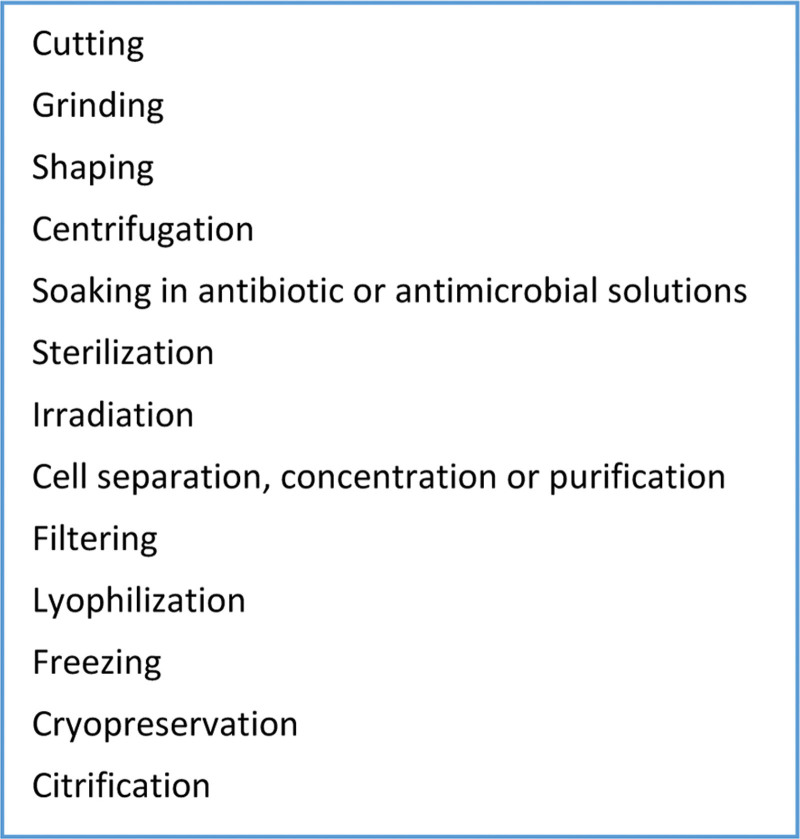
Manipulations *not* considered to be “substantial,” as defined in Annex I of Regulation (EC) no. 1394/2007 on advanced therapy medicinal products.

A cell-based therapy for type 1 diabetes could therefore fall into the classification of a sCTMPs or tissue engineered product depending upon the cell source used, their intended function, and if they are “substantially manipulated” (Figure 1).^22^

Increasingly complex novel therapies, particularly those incorporating multiple different cell types and a device, may not fit neatly into one of these classifications. The European regulations have sought to bridge this gap by advising that products should be considered under the most highly regulated tier of its component parts,

A product which may fall within the definition of a tissue engineered product and within the definition of a somatic cell therapy medicinal product shall be considered as a tissue engineered product.^22^

#### Committee for Advanced Therapies

To assist developers in navigating these classifications and to ensure that prospective products undergo the appropriate preclinical and clinical testing, the EMA established the Committee for Advanced Therapies (CAT). Here advice can be sought on the likely categorization of an investigational product free of charge at any time in the development process. All scientific opinions are then published and publicly available.^30^

#### Classification of Cellular Therapies for Type 1 Diabetes

With so many active research projects targeted at cellular therapies for diabetes, it may be surprising that between 2010 and 2020, the CAT has published scientific opinions on just 11 potential ATMPs aimed at type 1 diabetes.^31-38^ Of these, the majority (5) were classified as “sCMTPs,” just 2 were combined ATMPs, and 2 were felt not to fall within the remit of the ATMP framework—both of which contained human islets of Langerhans cells that had been insufficiently “manipulated” to bring them into the domain of ATMPs^33-38^ (Table 1). Strikingly when compared with recent research published from groups working at creating a regenerative medicine solution for type 1 diabetes, newer therapies seem likely to be far more complex than any of those which have been considered before.^18,39-42^

**TABLE 1.** Summary characteristics and classifications of products for type 1 diabetes published by the Committee for Advanced Therapies, 2010–2020

Year of decision	Summary characteristics	Classification given
2020	Insulin-producing pancreatic islet cells derived from human embryonic stem cells	sCTMP
2019	Allogeneic viable Wharton’s jelly–derived mesenchymal stem cells	ATMP
2020	Genetically modified *Lactococcus lactis* strain engineered to excrete human proinsulin and human IL-10	GTMP
2020	Adipose tissue–derived stem cells or –induced pluripotent stem cells transformed into insulin- and glucagon-releasing cells, cultured endothelial cells, and fibroblasts/fibrocytes	TEP + device = combined ATMP
2018	Allogeneic pancreatic islets encapsulated by an elastin-like recombiners	Not ATMP
2016	Autologous ex vivo expanded regulatory T lymphocytes	sCTMP
2016	Autologous ex vivo expanded polyclonal regulatory T cells	sCTMP
2015	Bone marrow–derived autologous nonhematopoietic stem cells suspended in a buffer	sCTMP
2013	Immunoprotected (alginate-encapsulated) porcine islet preparation	sCTMP + device = combined ATMP
2011	Human islets of Langerhans	Not ATMP
2010	Porcine beta cells and their accompanying endocrine cell populations embedded in an alginate matrix	sCTMP

ATMP, advanced therapy medicinal product; GTMP, gene therapy medicinal product; IL-10, interleukin 10; sCTMP, somatic cell therapy medicinal product; TEP, tissue engineered product.

#### The ATMP Bottleneck

As research into cellular and gene therapies expands it might be expected that an increasing number of products will come to market. However, there is a high attrition rate in the development of ATMPs and to date, despite hundreds of clinical trials, only 25 ATMPs for any disease process have made it successfully through the product development process and been granted market authorization.^23,43^ There are varied reasons for this apparent bottleneck. Working with cells and genetically modified organisms is costly and labor intensive and requires highly specialized personnel and laboratory conditions. It should not be surprising therefore that newly approved ATMPs often come with a significant price tag costing tens or hundreds of thousands of Euros.^44^ Of the few products that have made it to market, some have subsequently been withdrawn for commercial rather than medical reasons.^23,44,45^

In addition to these scientific and commercial challenges, the complex regulatory framework that such products are subjected to may be acting as a barrier to innovation. In a European survey of developers, regulatory challenges (34%) were cited more commonly than technical (30%) or scientific challenges (14%) in development of ATMPs.^45^

#### Regulation of ATMP Research and Development

To illustrate this regulatory complexity, we will consider the layers of legal and regulatory safeguards, which are applied or stripped away at each stage of the product development process.

##### Basic Scientific Research

Essential to creating a regenerative therapy for type 1 diabetes will be securing a reliable source of insulin-producing cells of a uniform quality. There are a variety of options for where these cells might originate from: deceased human donors, induced pluripotent stem cells, or xenogeneic cells have all been suggested.^18,39-41^ In addition, other cell types including those drawn from embryonic sources or peripheral blood might then be included to provide structural, immune protective, or vascularization functions.^18,39-41^ Each type of cellular component will have advantages and disadvantages from a scientific perspective, but they will also be subject to separate and specific regulatory requirements.

For example, if islet cells are collected from deceased human donors the first responsibility is to ensure the specific laws regarding organ donation of the country in which they originated are adhered to. Organs are then subject to the European Directive on the quality and safety of human organs intended for transplantation (Directive 2010/45/EU^5^). In contrast insulin-producing cells derived from stem cells would be subject to the tissues and cell directives (Directive 2004/23/EC^46^ and Directive 2006/17/EC^47^), which regulate the donation, procurement, testing, processing, preservation, storage, and distribution of human tissues and cells. Noninsulin secreting–supporting cells might be subject to the tissues and cells directives (Directive 2004/23/EC^46^ and Directive 2006/17/EC^47^) or the European Blood Directive (Directive 2002/98/EC^48^) depending on their source. Any cells that have undergone genetic modification will be subject to directives for genetically modified organisms (Directive 2001/18/EC^49^ and Directive 2009/41/EC^50^). A product that combines multiple cellular sources may therefore need to adhere to requirements from several different regulations.

##### Clinical Trials

Once a preparation has been formulated, those that are promising move into clinical trials wherein clinical researchers and commercial developers must adhere to the Clinical Trials Regulations (Regulation EU No 536/2014^51^) and Directives on good clinical practice (Directive 2001/20/EC^52^ and Directive 2005/28/EC^53^). In addition, there are those for good manufacturing practice (Directive 2003/94/EC^54^) governing the safety and quality standards for laboratories and manufacturing facilities creating treatments intended for human use.

##### Market Authorization

It is only at the market authorization stage that the specific regulations for ATMPs come into full action (Regulation No. 1394/2007 EC^55^) as well as Directive 2009/120/EC relating to medicinal products for human use as regard ATMPs.^50^ Should the product be used in pediatric populations further regulations aimed specifically at medicinal products for pediatric use [Regulation (EC) No 1901/2006^56^ and Regulation (EC) No 1902/2006^57^] must be adhered to and specific pediatric investigation plans, which outline the necessary studies needed for drugs used in children, may be required.

##### Pharmacovigilance

Finally, once a product is authorized, it remains subject to pharmacovigilance Regulations and Directives [Regulation (EU) No 1235/2010^58^ and Directive 2010/84/EU^59^].

#### Regulating Medical Devices—Impacts for Combined Products

As alluded to earlier, some novel therapies in development do not just contain cellular components but also include components that meet the definition of “medical devices.” For example, decellularized tissues or completely synthetic materials may be used as scaffolds to protect or aid aggregation of cells, 3D printing techniques, and equipment, or the entire “bioartifical pancreas” might be encapsulated within a device to allow for ease of insertion and removal.^18,39-42^ The devices used in these “combined ATMPs” will be required to demonstrate that they conform to the medical devices regulations (Regulation 2017/745^60^).

In a significant difference to the regulation of pharmaceutical or cellular therapies, the EMA does not act as a central authorizing agency for medical devices. Instead, national “notified bodies” assess products manufactured in each member state. These bodies conduct a conformity assessment of any proposed new product and once satisfied issue a Conformité Européenne mark before the device is used in patients. For devices considered “high-risk,” the EMA provides scientific advice and oversight, but the bulk of the assessments of new devices are devolved.^61^

Therefore, for combined ATMPs, the EMA would take into consideration the relevant notified bodies assessment of the device component(s) before issuing an assessment and market authorization of the ATMP product as a whole. So, the final assessment of a combined ATMP would remain the remit of the EMA. However, when multiple agencies are involved, the possibility emerges of heterogeneous interpretation and application of rules and standards.

#### Criticisms of Medical Device Regulation

High-profile scandals involving breast implants and metal-on-metal hip replacements have drawn attention to the regulation of medical devices resulting in the EU regulations being updated in 2017 (although only coming into force in 2021).^60,62^ In these cases, products were withdrawn in different jurisdictions at different stages resulting in some patients in both France and the United Kingdom being exposed to products, which had already been banned in the United States.^62,63^ This has led to specific criticism of the devolved European model in comparison to the United States where the Food and Drug Administration assesses *all* medical devices centrally.^62^ The updated European regulations have increased standards for testing of devices before coming to market and enhanced the sanctions regime. However, some critics still argue that this “non-harmonized” style of regulation is both complicated and burdensome for manufacturers and insufficiently protects consumers with potentially disastrous consequences.^62^

### The Regulatory Burden

Regulation of frontier medical science operates under a constant tension—on the one hand facilitating and promoting innovation, and on the other securing protection from harm for recipients of novel treatments. The EMA has recognized that there is a need to “ensure that the regulatory framework supports—and not hinders—the development of ATMPs.”^64^ However, they have also issued stark warnings about the risks of unproven cell-based therapies, which can carry “serious, sometimes fatal, side effects including infections, unwanted immune reactions [and] tumor formation”^65^ and explicitly warned patients against accepting cell-based therapies, which have not been proven effective in formal clinical trials.

Our analysis shows that for products that use the most complex combinations of cellular and noncellular device components over 20 different EU directives may be engaged over the course of development (**Table S1, SDC**, http://links.lww.com/TP/C878). It is important to note that these various legal instruments will not all apply to the finished product, many will apply only at certain phases of the iterative development process. However, there is a cumulative burden in ensuring that each regulation’s specific requirements are adhered to at the necessary stages. This requires an extremely detailed understanding of the law on the part of developers, which may be beyond the capability and resources of smaller organizations.

Predictably, there may be attempts to circumvent this regulatory burden when possible. Under the existing legislation, the “hospital exemption” criteria allow ATMPs, which are produced in a single hospital or clinic to be excluded from the ATMP regulatory framework.^55^ This facilitates the production of targeted cell–based treatments to be used only in individual patients at specialist centers. However, it has also stimulated concern that there is the potential for therapies to be offered that have not meet the required safety standards and put patients at risk.^65,66^

#### Regulating Products Incorporating Cells of Xenogeneic Origin

Generating a plentiful supply of reliable insulin–producing cells has been 1 of the most challenging scientific hurdles to overcome in formulating novel beta–cell therapies. One solution, which has been proposed, is that cells from genetically modified animals could provide a relatively unlimited supply of islet cells for an ATMP. Utilizing xenogeneic cells as a therapy for type 1 diabetes has been attempted as far back as the 1990s.^67^ Although to date a successful product has not been brought to market, the EMA has provided preemptive guidance on the standards for the use of animal cells in Xenogeneic Medicinal Products^68^ and stated that *any* products “containing or consisting of [*viable*] animal cells or tissues to be administered to humans will always be considered as ATMPs.”^69^ Beyond this, however, the permissibility of xenotransplantation or the use of xenogeneic cells in medicinal products is a matter devolved to individual member states with different approaches being taken across the block.^70^ Therefore, if a xenogeneic product were to be brought to market under the current regime, there is no guarantee it would be uniformly regulated or accessible across Europe. Given the recent advances in whole organ xenotransplantation,^71,72^ the EU will need to consider urgently if further regulations or specific amendments are needed to harmonize the approach.

#### Nomenclature, Classification, and Clinical Consequences

As demonstrated earlier, classification can have a significant impact on regulatory oversight. However, there are also clinical consequences. In 2011, the EMA was asked to consider if the preparation of cells for use in islet cell transplantation met the threshold for substantial manipulation to be included in ATMP regulation. They found that as the cells involved were “minimally manipulated” and “intended to be used for the same essential function in the recipient and the donor, that is, pancreatic function”^35^ that they did not fall into the more complicated regulatory framework of ATMPs and remained regulated in the same manner as whole organ transplantation.

In contrast, the Food and Drug Administration in the United States determined in 2015 that, due to the processing of cells, islet cell transplantation fell under the regulation of biologic therapies, which are broadly equivalent to those for ATMPs. This has led to what 1 commentator describes as “the demise of islet allotransplantation in the United States”^73^ with only 11 recipients receiving islet allotransplantation between 2016 and 2019, and all of these under the auspices of a clinical trial as an investigational product. Therefore, definitions of manipulation, nomenclature, and classification can significantly impact the clinical availability of even well-established treatments. The impact upon novel and innovative ones could prove pivotal in determining a treatment’s availability and ultimate success.^74^

### The Future of Regulation

There are already moves underway to shift the regulatory paradigm. To simplify the existing network of regulations, the European Commission is currently developing a new proposed regulation, which would replace the existing directives covering blood, cells, and tissues. This new unified regulation would also extend the Commission’s regulatory oversight to previously unregulated substances such as human breastmilk ensuring the safety and quality of all “substances of human origin” (SoHO).^75^ The expressed intention is to build a

future-proof and robust framework which better protects donors, recipients treated by transfusion, and transplantation … [and] that fosters innovation in this crucial biotech sector.^75^

However, it is not just regulatory complexity that is of concern. As we move rapidly from scientific theory to clinical practice, regenerative medicine products are likely to encounter challenges surrounding 1 of the most significant current debates in medicine. That of equality of access. There are known disparities in the uptake of technological treatments for diabetes with the subsequent impacts on patient outcomes felt disproportionately by some marginalized groups.^76^ The WHO have highlighted disparities in access to cellular treatments between high and low and middle income countries as a priority for resolution.^21^ To allow this area to realize its full potential these disparities must be corrected. To that end, regulations will need to begin to consider the wider legal and social context in which these products will be marketed.

## CONCLUSION

There can be little doubt that the future of the treatment of type 1 diabetes lies in regenerative medicine, with a bioartificial pancreas providing our most compelling chance at creating a truly bioartificial organ in the near future. Centralized EU regulations have gone a significant way to aligning the approach to novel cell-based therapies across Europe. However, despite this the legal landscape for products containing multiple cell sources, genetic modifications, and in combination with medical devices remains remarkably complicated. This may form an obstacle to innovation and have significant implications for clinical use. In addition, the use of cells of xenogeneic origin represents an area of European law in which there is very little consensus. As we reach further into the frontiers of cellular therapy, there are challenges but also opportunities to rethink how we regulate highly innovative medical procedures. Promoting regulatory convergence to facilitate development and access to these novel products for patients in all regions of the world is an important first step.

## ACKNOWLEDGMENTS

The authors acknowledge the valuable contributions of their collaborators Dr Eline Bunnik, Dr Emma Massey, and Dide De Jongh. In addition, they acknowledge the mentorship of Professor Alex Ruck-Keene and Professor Kristof Van Assche and the support of the VANGUARD Consortium.

## VANGUARD Consortium:

Ekaterine Berishvili, Laura Mar Fonseca, Fanny Lebreton, Kevin Bellofatto, Juliette Bignard, Reine Hanna, Victor Galvan, University of Geneva, Department of Surgery, Geneva, Switzerland.

Jochen Seissler, Leila Wolf-van Buerck, Mohsen Honarpisheh, Yichen Zhang, Yutian Lei, Monika Pehl, Diabetes Centre, Ludwig-Maximilians University Munich, Germany.

Antonia Follenzi, Chiara Borsotti, Simone Merlin, Department of Health Sciences, University of Piemonte Orientale, Novara, Italy.

Lorenzo Piemonti, Antonio Citro, Silvia Pellegrini, IRCCS Ospedale San Raffaele, Diabetes Research Institute, Milano, Italy.

Olivier Thaunat, Morgane Fouche, Lyon Claude Bernard University, Department of Transplantation, Nephrology and Clinical Immunology, Lyon, France.

Devi Mey, Chiara Parisotto, Giovanna Rossi, European Society for Organ Transplantation, Padova, Italy.

Patrick Kugelmeier, Petra Wolint, Markus Mühlemann, Karolina Pal-Kutas, Kugelmeiers AG, Erlenbach, Switzerland.

Marco Cavallaro, Julia Götz, Jeanette Müller, Accelopment Switzerland Ltd.

## Supplementary Material

## References

[1] Pathak V Pathak NM O’Neill CL. Therapies for type 1 diabetes: current scenario and future perspectives. Clin Med Insights Endocrinol Diabetes. 2019;12:117955141984452. 10.1177/1179551419844521 PMC6501476 31105434

[2] McCrimmon RJ Henry RR. SGLT inhibitor adjunct therapy in type 1 diabetes. Diabetologia. 2018;61:2126–2133. 30132030 10.1007/s00125-018-4671-6 PMC6133151

[3] European Parliament. Directive 2001/83/EC relating to medicinal products for human use. Available at https://eur-lex.europa.eu/homepage.html.

[4] European Parliament. Directive 98/79/EC of the European Parliament and of the Council of 27 October 1998 on in vitro diagnostic medical devices. Available at https://eur-lex.europa.eu/homepage.html.

[5] European Parliament. Directive 2010/45/EU of the European Parliament and of the Council of 7 July 2010 on standards of quality and safety of human organs intended for transplantation. Available at https://eur-lex.europa.eu/homepage.html.

[6] VANGUARD. Available at https://vanguard-project.eu/. Accessed March 7, 2023.

[7] ViaCyte. Available at https://viacyte.com/. Accessed March 7, 2023.

[8] VERTEX. R&D pipeline. Available at https://www.vrtx.com/our-science/pipeline/. Accessed March 7, 2023.

[9] Gruessner AC Gruessner RWGT. International pancreas transplant registry report—a review. Transplant Proc. 2022;2022:1918–1943. 10.1016/j.transproceed.2022.03.059 35970624

[10] Berney T Andres A Bellin MD; International Islet Transplant Centers. International islet transplant centers: a worldwide survey of activities and practices in clinical islet of langerhans transplantation. Transpl Int. 2022;35:10507. 36033644 10.3389/ti.2022.10507 PMC9402897

[11] Wojtusciszyn A Branchereau J Esposito L; TREPID Group. Indications for islet or pancreatic transplantation: statement of the TREPID working group on behalf of the Société francophone du diabète (SFD), Société francaise d’endocrinologie (SFE), Société francophone de transplantation (SFT) and Société française de néphrologie—dialyse—transplantation (SFNDT). Diabetes Metab. 2019;45:224–237. 30223084 10.1016/j.diabet.2018.07.006

[12] Dholakia S Royston E Quiroga I. The rise and potential fall of pancreas transplantation. Br Med Bull. 2017;124:171–179. 29088319 10.1093/bmb/ldx039

[13] Gruessner AC Gruessner RWG. Pancreas transplantation of US and non-US cases from 2005 to 2014 as reported to the United Network for Organ Sharing (UNOS) and the International Pancreas Transplant Registry (IPTR). Rev Diabet Stud. 2016;13:35–58. 26982345 10.1900/RDS.2016.13.35 PMC5291181

[14] Cimen SG Cimen S Kessaris N. Challenges of pancreas transplantation in developing countries, exploring the Turkey example. World J Transplant. 2019;9:158–164. 31966972 10.5500/wjt.v9.i8.158 PMC6960118

[15] Manrique A Jimenez C Lopez RM. Relaparotomy after pancreas transplantation: causes and outcomes. Transplant Proc. 2009;41:2472–2474. 19715955 10.1016/j.transproceed.2009.06.165

[16] Collaborative Islet Transplant Registry. Eleventh allograft report. 2022. Available at https://citregistry.org/system/files/11th%20Allograft%20report%20May%2031%202022.pdf. Accessed March 7, 2023.

[17] Caiazzo R Vantyghem MC Raverdi V. Impact of procedure-related complications on long-term islet transplantation outcome. Transplantation. 2015;99:979–984. 25393157 10.1097/TP.0000000000000458

[18] Berney T Wassmer CH Lebreton F. From islet of langerhans transplantation to the bioartificial pancreas. Presse Med. 2022;51:104139. 36202182 10.1016/j.lpm.2022.104139

[19] Hering BJ Ballou CM Bellin MD. Factors associated with favourable 5 year outcomes in islet transplant alone recipients with type 1 diabetes complicated by severe hypoglycaemia in the Collaborative Islet Transplant Registry. Diabetologia. 2023;66:163–173. 36201044 10.1007/s00125-022-05804-4 PMC10355148

[20] Bellin MD Dunn TB. Transplant strategies for type 1 diabetes: whole pancreas, islet and porcine beta cell therapies. Diabetologia. 2020;63:2049–2056. 32894315 10.1007/s00125-020-05184-7

[21] World Health Organization. WHO considerations on regulatory convergence of cell and gene therapy products. Draft for public consultation. Available at https://cdn.who.int/media/docs/default-source/biologicals/ecbs/who-public-consultation_cgtp-white-paper_16_dec_2021.pdf?sfvrsn=18f6c549_5. Accessed April 12, 2023.

[22] European Parliament. Regulation (EC) No 1394/2007 EC (on advanced therapy medicinal products and amending Directive 2001/83/EC (medicinal products for human use) and Regulation (EC) No 726/2004 (on procedures for the authorisation and supervision of medicines for human and veterinary use and establishing the European Medicines Agency); 2007.

[23] Cornwall T Taylor S Williams E. Advanced therapies: innovation, regulation and patentability in the UK. Biosci Law Rev. 2021;18:11.

[24] NHS England. First baby receives life-saving gene therapy on NHS. Available at https://www.england.nhs.uk/2023/02/first-baby-receives-life-saving-gene-therapy-on-nhs/. Accessed May 17, 2023.

[25] Green A Hede SM Patterson CC. Type 1 diabetes in 2017: global estimates of incident and prevalent cases in children and adults. Diabetologia. 2021;64:2741–2750. 34599655 10.1007/s00125-021-05571-8 PMC8563635

[26] International Diabetes Federation. Diabetes in Europe—2021. Available at https://www.mepinterestgroupdiabetes.eu/wp-content/uploads/2021/11/IDF-Atlas-Factsheet-2021_EUR.pdf. Accessed March 28, 2023. 35914061

[27] US National Library of Medicine. Clinicaltrials.gov. Available at https://clinicaltrials.gov/ct2/results?cond=Diabetes+Mellitus%2C+Type+1&term=CELL+THERAPY+&cntry=&state=&city=&dist=. Accessed May 26, 2023. 10.1080/15360280801989377 28792816

[28] European Parliament. Directive 2001/83/EC of the European Parliament and of the Council of 6 November 2001 on the community code relating to medicinal products for human use; 2001.

[29] European Medicines Agency. Advanced therapy medicinal products: overview. Available at https://www.ema.europa.eu/en/human-regulatory/overview/advanced-therapy-medicinal-products-overview. Accessed April 14, 2023.

[30] European Medicines Agency. Scientific recommendations on classification of advanced therapy medicinal products. Available at https://www.ema.europa.eu/en/human-regulatory/marketing-authorisation/advanced-therapies/advanced-therapy-classification/scientific-recommendations-classification-advanced-therapy-medicinal-products. Accessed June 27, 2023.

[31] European Medicines Agency. Scientific recommendations on classification of advanced therapy medicinal products. Available at https://www.ema.europa.eu/documents/other/scientific-recommendations-classification-advanced-therapy-medicinal-products_en.xlsx. Accessed November 4, 2022.

[32] European Medicines Agency. Scientific recommendations on classification of advanced therapy medicinal products (April 2019–December 2020). Available at https://www.ema.europa.eu/documents/other/scientific-recommendations-classification-advanced-therapy-medicinal-products-april-2019-december_en.xlsx. Accessed November 4, 2022.

[33] European Medicines Agency. Scientific recommendation on classification of advanced therapy medicinal products: allogeneic pancreatic islets encapsulated by an elastin-like recombinamers. Available at https://www.ema.europa.eu/en/documents/report/scientific-recommendation-classification-advanced-therapy-medicinal-products-allogeneic-pancreatic_en.pdf. Accessed October 20, 2022.

[34] European Medicines Agency. Scientific recommendation on classification of advanced therapy medicinal products: alginate encapsulated porcine pancreatic islet cells. Available at https://www.ema.europa.eu/en/documents/report/scientific-recommendation-classification-advanced-therapy-medicinal-products-alginate-encapsulated_en.pdf. Accessed October 21, 2022.

[35] European Medicines Agency. Scientific recommendation on classification of advanced therapy medicinal products: suspension containing human islets of Langerhans, autologous or allogeneic. Available at https://www.ema.europa.eu/en/documents/report/scientific-recommendation-classification-advanced-therapy-medicinal-products-human-islets-langerhans_en.pdf. Accessed October 21, 2022.

[36] European Medicines Agency. Scientific recommendation on classification of advanced therapy medicinal products: suspension of autologous regulatory T lymphocytes. Available at https://www.ema.europa.eu/en/documents/report/scientific-recommendation-classification-advanced-therapy-medicinal-products-autologous-ex-vivo_en.pdf. Accessed April 21, 2022.

[37] European Medicines Agency. Scientific recommendation on classification of advanced therapy medicinal products: autologous ex vivo expanded polyclonal CD4+CD25+CD127lo/-FOXP3+ regulatory T cells. Available at https://www.ema.europa.eu/en/documents/report/scientific-recommendation-classification-advanced-therapy-medicinal-products-autologous-ex-vivo_en.pdf. Accessed April 21, 2022.

[38] European Medicines Agency. Scientific recommendation on classification of advanced therapy medicinal products: bone marrow-derived autologous non-hematopoietic stem cells. Available at https://www.ema.europa.eu/en/documents/report/scientific-recommendation-classification-advanced-therapy-medicinal-products-bone-marrow-derived_en-3.pdf. Accessed April 21, 2022.

[39] Photiadis SJ Gologorsky RC Sarode D. The current status of bioartificial pancreas devices. ASAIO. 2021;67:370–381. 10.1097/MAT.0000000000001252 PMC7996235 32826394

[40] Wassmer CH Lebreton F Bellofatto K; VANGUARD Consortium. Bio-engineering of pre-vascularized islet organoids for the treatment of type 1 diabetes. Transpl Int. 2022;35:10214. 35185372 10.3389/ti.2021.10214 PMC8842259

[41] Hanna R Berishvili E. Advances and challenges of endocrine pancreas bioengineering. Curr Opin Endocr Metab Res. 2022;23:100320.

[42] Wassmer CH Lebreton F Bellofatto K. Generation of insulin-secreting organoids: a step toward engineering and transplanting the bioartificial pancreas. Transpl Int. 2020;33:1577–1588. 32852858 10.1111/tri.13721 PMC7756715

[43] Iglesias-Lopez C Agustí A Vallano A. Current landscape of clinical development and approval of advanced therapies. Mol Ther Methods Clin Dev. 2021;23:606–618. 34901306 10.1016/j.omtm.2021.11.003 PMC8626628

[44] Ronco V Dilecce M Lanati E. Price and reimbursement of advanced therapeutic medicinal products in Europe: are assessment and appraisal diverging from expert recommendations? J Pharm Policy Pract. 2021;14:30. 33741076 10.1186/s40545-021-00311-0 PMC7980570

[45] Ten Ham RMT Hoekamn J Hovels AM. Challenges in advanced therapy medicinal product development: a survey among companies in Europe. Mol Ther Methods Clin Dev. 2018;11:121–130. 30456217 10.1016/j.omtm.2018.10.003 PMC6234262

[46] European Parliament. Directive 2004/23/EC, also known as the European Tissues and Cells Directive, covering standards for donation, procurement and testing, processing, preservation, storage and distribution of human tissues and cells. Available at https://eur-lex.europa.eu/homepage.html.

[47] European Parliament. Directive 2006/17/EC of 8 February 2006 implementing Directive 2004/23/EC of the European Parliament and of the Council as regards certain technical requirements for the donation, procurement and testing of human tissues and cells. Available at https://eur-lex.europa.eu/homepage.html.

[48] European Parliament. Directive 2002/98/EC (amending Directive 2001/83/EC) of January 2003 sets standards of quality and safety for the collection, testing, processing, storage and distribution of human blood and blood components. Available at https://eur-lex.europa.eu/homepage.html.

[49] European Parliament. Directive 2001/18/EC of the European Parliament and of the Council of 12 March 2001 on the deliberate release into the environment of genetically modified organisms and repealing Council Directive 90/220/EEC. Available at https://eur-lex.europa.eu/homepage.html.

[50] European Parliament. Directive 2009/41/EC of the European Parliament on the contained use of genetically modified micro-organisms. Available at https://eur-lex.europa.eu/homepage.html.

[51] European Parliament. Regulation (EU) No 536/2014 of the European Parliament and of the Council of 16 April 2014 on clinical trials on medicinal products for human use, and repealing Directive 2001/20/EC. Available at https://eur-lex.europa.eu/homepage.html.

[52] European Parliament. Directive 2001/20/EC of April 2001—lays down approximation of the laws, regulations and administrative provisions of the Member States relating to the implementation of good clinical practice in the conduct of clinical trials on medicines for human use. Available at https://eur-lex.europa.eu/homepage.html. 16276663

[53] European Parliament. Directive 2005/28/EC of April 2005 laying down principles and detailed guidelines for good clinical practice as regards investigational medicinal products for human use, as well as the requirements for authorisation of the manufacturing or importation of such products. Available at https://eur-lex.europa.eu/homepage.html.

[54] European Parliament. Directive 2003/94/EC of October 2003 laying down the principles and guidelines of good manufacturing practice in respect of medicinal products for human use and investigational medicinal products for human use. Available at https://eur-lex.europa.eu/homepage.html.

[55] European Parliament. Regulation (EC) No 1394/2007 of the European Parliament and of the Council of 13 November 2007 on advanced therapy medicinal products and amending Directive 2001/83/EC and Regulation (EC) No 726/2004. No 1394/2007. Available at https://eur-lex.europa.eu/homepage.html.

[56] European Parliament. Regulation (EC) No 1901/2006 of the European Parliament and of the Council of 12 December 2006 on medicinal products for paediatric use and amending Regulation (EEC) No 1768/92, Directive 2001/20/EC, Directive 2001/83/EC and Regulation (EC) No 726/2004. Available at https://eur-lex.europa.eu/homepage.html.

[57] European Parliament. Regulation (EC) No 1902/2006 of the European Parliament and of the Council of 20 December 2006 amending Regulation 1901/2006 on medicinal products for paediatric use. Available at https://eur-lex.europa.eu/homepage.html.

[58] European Parliament. Regulation (EU) No 1235/2010 of 15 December 2010 amending, as regards pharmacovigilance of medicinal products for human use, Regulation (EC) No 726/2004 laying down Community procedures for the authorisation and supervision of medicinal products for human and veterinary use and establishing a European Medicines Agency, and Regulation (EC) No 1394/2007 on advanced therapy medicinal products. Available at https://eur-lex.europa.eu/homepage.html.

[59] European Parliament. Directive 2010/84/EU of 15 December 2010 amending, as regards pharmacovigilance, Directive 2001/83/EC on the Community code relating to medicinal products for human use. Available at https://eur-lex.europa.eu/homepage.html.

[60] European Parliament. Regulation (EU) 2017/745 of the European Parliament and of the Council of 5 April 2017 on medical devices, amending Directive 2001/83/EC, Regulation (EC) No 178/2002 and Regulation (EC) No 1223/2009 and repealing Council Directives 90/385/EEC and 93/42/EEC. Available at https://eur-lex.europa.eu/homepage.html.

[61] European Medicines Agency. Medical devices. Available at https://www.ema.europa.eu/en/human-regulatory/overview/medical-devices#high-risk-medical-devices-section. Accessed April 17, 2023.

[62] Jarman H Rozenblum S Huang TJ. Neither protective nor harmonized: the crossborder regulation of medical devices in the EU. Health Econ Policy Law. 2021;16:51–63. 32631465 10.1017/S1744133120000158

[63] Yukhananov A. Insight: FDA warned PIP on breast implant safety in 2000. *Reuters*. Available at https://www.reuters.com/article/us-breastimplants-fda/insight-fda-warned-pip-on-breast-implant-safety-in-2000-idUSTRE7BQ03M20111228. Accessed May 26, 2023.

[64] European Medicines Agency. European Commission-DG Health and Food Safety and European Medicines Agency action plan on ATMPs. Available at https://www.ema.europa.eu/documents/other/european-commission-dg-health-food-safety-european-medicines-agency-action-plan-advanced-therapy_en-0.pdf. Accessed October 28, 2023.

[65] European Medicines Agency. EMA warns against using unproven cell-based therapies. Available at https://www.ema.europa.eu/documents/public-statement/ema-warns-against-using-unproven-cell-based-therapies_en.pdf. Accessed October 20, 2022.

[66] Food and Drug Administration. FDA warns about stem cell therapies. Available at https://www.fda.gov/consumers/consumer-updates/fda-warns-about-stem-cell-therapies. Accessed May 26, 2023.

[67] Groth C. Transplantation of porcine fetal pancreas to diabetic patients. Lancet. 1994;345:735–1404. 10.1016/s0140-6736(95)90910-9 7885159

[68] European Medicines Agency. Guideline on xenogeneic cell-based medicinal products. Available at https://www.ema.europa.eu/en/documents/scientific-guideline/guideline-xenogeneic-cell-based-medicinal-products_en.pdf. Accessed October 20, 2022.

[69] European Medicines Agency. Reflection paper on classification of advanced therapy medicinal products. Available at https://www.ema.europa.eu/en/documents/scientific-guideline/reflection-paper-classification-advanced-therapy-medicinal-products_en-0.pdf. Accessed October 20, 2022.

[70] Council of Europe. Report on the state of the art in the field of xenotransplantation. Available at https://www.coe.int/t/dg3/healthbioethic/Activities/06_Xenotransplantation_en/XENO(2003)1_SAR.pdf. Accessed October 27, 2022.

[71] Porrett PM Orandi BJ Kumar V. First clinical-grade porcine kidney xenotransplant using a human decedent model. Am J Transplant. 2022;22:1037–1053. 35049121 10.1111/ajt.16930

[72] Reardon S. First pig-to-human heart transplant: what can scientists learn? Nature. 2022;601:305–306. 35031782 10.1038/d41586-022-00111-9

[73] Witkowski P Philipson LH Kaufman DB; “Islets for US” Collaborative. The demise of islet allotransplantation in the United States: a call for an urgent regulatory update. Am J Transplant. 2021;21:1365–1375. 33251712 10.1111/ajt.16397 PMC8016716

[74] Piemonti L Andres A Casey J. US Food and Drug Administration (FDA) panel endorses islet cell treatment for type 1 diabetes: a pyrrhic victory? Transpl Int. 2021;34:1182–1186. 34048106 10.1111/tri.13930

[75] European Commission. Questions and answers on the proposal for a new legislation on blood, tissues, and cells. Available at https://ec.europa.eu/commission/presscorner/detail/en/qanda_22_4404. Accessed March 2, 2023.

[76] Auzanneau M Rosenbauer J Maier W. Heterogeneity of access to diabetes technology depending on area deprivation and demographics between 2016 and 2019 in Germany. J Diabetes Sci Technol. 2021;15:1059–1068. 34253084 10.1177/19322968211028608 PMC8442190

