# Supplementary material for: Legal and Regulatory Challenges for Emerging Regenerative Medicine Solutions for Diabetes
Source: Transplantation. 2023 Sep 26;108(5):1072–9. doi: 10.1097/TP.0000000000004797 (PMC11042516; doi:10.1097/TP.0000000000004797)
Supplement: Supplementary file 1 [file tpa-108-1072-s001.pdf]

Supplemental Digital Content

Table S1. Summary of European Union Regulations and Directives which may be applicable to a beta-cell based regenerative medicine product and its components.

	**EU regulations and directives**
**Tissues and cells**	Directive 2004/23/EC, also known as the European Tissues and Cells Directive, covering standards for donation, procurement and testing, processing, preservation, storage and distribution of human tissues and cells<br>Directive 2006/17/EC of 8 February 2006 implementing Directive 2004/23/EC of the European Parliament and of the Council as regards certain technical requirements for the donation, procurement and testing of human tissues and cells
**Blood components**	Directive 2002/98/EC (amending Directive 2001/83/EC) of January 2003 sets standards of quality and safety for the collection, testing, processing, storage and distribution of human blood and blood components.
**Genetically modified organisms**	Directive 2009/41/EC of the European Parliament on the contained use of genetically modified micro-organisms<br>Directive 2001/18/EC of the European Parliament on the deliberate release into the environment of genetically modified organisms
**Use of animals**	Directive 2010/63/EU of the European Parliament and of the Council of 22 September 2010 on the protection of animals used for scientific purposes
**Transplantation**	Directive 2010/45/EU of the European Parliament and of the Council of 7 July 2010 on standards of quality and safety of human organs intended for transplantation
**Clinical trials**	Regulation (EU) No 536/2014 of the European Parliament and of the Council of 16 April 2014 on clinical trials on medicinal products for human use, and repealing Directive 2001/20/EC<br>Directive 2001/20/EC of April 2001 - lays down approximation of the laws, regulations and administrative provisions of the Member States relating to the implementation of good clinical practice in the conduct of clinical trials on medicines for human use<br>Directive 2003/94/EC of October 2003 laying down the principles and guidelines of good manufacturing practice in respect of medicinal products for human use and investigational medicinal products for human use
**Good laboratory practice**	Directive 2004/10/EC of the European Parliament and of the Council of 11 February 2004 on the harmonisation of laws, regulations and administrative provisions relating to the application of the principles of good laboratory practice and the verification of their applications for tests on chemical substances
**Good manufacturing practice**	Regulation (EU) No 1252/2014 of 28 May 2014 supplementing Directive 2001/83/EC of the European Parliament and of the Council with regard to principles and guidelines of good manufacturing practice for active substances for medicinal products for human use<br>Directive 2003/94/EC of October 2003 laying down the principles and guidelines of good manufacturing practice in respect of medicinal products for human use and investigational medicinal products for human use
**Good clinical practice**	Directive 2001/20/EC of April 2001 - lays down approximation of the laws, regulations and administrative provisions of the Member States relating to the implementation of good clinical practice in the conduct of clinical trials on medicines for human use

	Directive 2005/28/EC of April 2005 laying down principles and detailed guidelines for good clinical practice as regards investigational medicinal products for human use, as well as the requirements for authorisation of the manufacturing or importation of such products
**Advanced therapy medicinal products**	Regulation (EC) No 1394/2007 EC on advanced therapy medicinal products and amending Directive 2001/83/EC (medicinal products for human use) and Regulation (EC) No 726/2004 (on procedures for the authorisation and supervision of medicines for human and veterinary use and establishing the European Medicines Agency)<br><br>Directive 2009/120/EC relating to medicinal products for human use as regards advanced therapy medicinal products
**Medicines for human use**	Directive 2001/83/EC relating to medicinal products for human use
**Medical devices**	Regulation (EU) 2017/745 of the European Parliament and of the Council of 5 April 2017 on medical devices, amending Directive 2001/83/EC, Regulation (EC) No 178/2002 and Regulation (EC) No 1223/2009 and repealing Council Directives 90/385/EEC and 93/42/EEC
**Pharmacovigilance**	Regulation (EU) No 1235/2010 of 15 December 2010 amending, as regards pharmacovigilance of medicinal products for human use, Regulation (EC) No 726/2004 laying down Community procedures for the authorisation and supervision of medicinal products for human and veterinary use and establishing a European Medicines Agency, and Regulation (EC) No 1394/2007 on advanced therapy medicinal products<br><br>Directive 2010/84/EU of 15 December 2010 amending, as regards pharmacovigilance, Directive 2001/83/EC on the Community code relating to medicinal products for human use
**Paediatric use**	Regulation (EC) No 1901/2006 of the European Parliament and of the Council of 12 December 2006 on medicinal products for paediatric use and amending Regulation (EEC) No 1768/92, Directive 2001/20/EC, Directive 2001/83/EC and Regulation (EC) No 726/2004<br><br>Regulation (EC) No 1902/2006 of the European Parliament and of the Council of 20 December 2006 amending Regulation 1901/2006 on medicinal products for paediatric use
**Data protection**	Regulation (EU) 2016/679 of the European Parliament and of the Council of 27 April 2016 on the protection of natural persons with regard to the processing of personal data and on the free movement of such data
